# Biomechanical study of a novel self-locking plate system for anterior cervical fixation

**DOI:** 10.1186/s13018-014-0120-5

**Published:** 2014-11-27

**Authors:** Lifeng Lao, Qianyi Li, Guibin Zhong, Chao Song, Yuanchao Li, Mingze Xu, Zude Liu

**Affiliations:** Department of Orthopaedic Surgery, Ren Ji Hospital, School of Medicine, Shanghai Jiao Tong University, Shanghai, China; School of Mechanical Engineering, Shanghai Jiao Tong University, Shanghai, China

**Keywords:** Cervical fixation, Self-locking plate, Biomechanics, DePuy plate

## Abstract

**Background:**

Anterior cervical plate had developed continuously, and this study aimed to assess the biomechanics of a novel self-locking plate system for anterior cervical fixation designed by ourselves.

**Methods:**

Twelve anterior cervical plates (i.e., six novel plates and six DePuy plates) were subjected to a pull-out test and a fatigue test. In addition, 12 C1-T1 cervical spine specimens underwent anterior cervical corpectomy and C5 fusion using six novel plates and six DePuy plates. Pre- and postoperative range of motion, load–displacement, axial stiffness, torque, and twisting stiffness were compared.

**Results:**

No differences in maximum pull-out force, relative displacement, or energy absorption were observed between the DePuy plates and the novel plates (*P* >0.05). The novel plate system could bear an average of 5.6 × 10^5^ times of loading, while the DePuy plate could bear 5.4 × 10^5^ times of loading. The fatigue strengths of the new plate system and the DePuy plate were 490.75 and 485.86 MPa, respectively. No differences in fatigue life or strength were observed between the two types of plates. Cervical spine stability increased significantly after internal fixation. No differences in range of motion, load–displacement, axial stiffness, torque, or twisting stiffness were observed between the novel self-locking plate and the DePuy plate (*P* >0.05).

**Conclusions:**

Compared to the DePuy plate, the novel anterior cervical self-locking plate system described here has good strength and fastening ability, allowing it to provide sufficient biomechanical stability. Further clinical assessment of this system is needed.

## Introduction

Cervical damage caused by trauma, deformities, degeneration, or tumor treatment is frequently reconstructed using anterior cervical plate fixation, which enhances postoperative cervical spine stability, reduces the incidence of pseudoarthrosis, and reduces cervical kyphosis [1,2]. Since the description of the anterior approach for cervical discectomy and fusion by Smith and Robinson [3] in 1958, anterior cervical procedures have become quite common with generally good clinical results. As the anterior cervical internal fixation system developed, more elderly patients underwent cervical fusion surgery. However, postoperative cervical screw collapse, pull-out, and breakage frequently occur due to osteoporosis [4,5]. We designed and developed a novel anterior cervical self-locking plate system based on a currently available cervical plate. The biomechanics of the self-locking plate were analyzed in comparison to the anterior cervical DePuy plate (Johnson & Johnson Company, NJ, USA), providing a basis for the clinical application of this novel plate.

## Materials and methods

### Structure of a novel self-locking plate system for anterior cervical fixation

**Figure 1:**
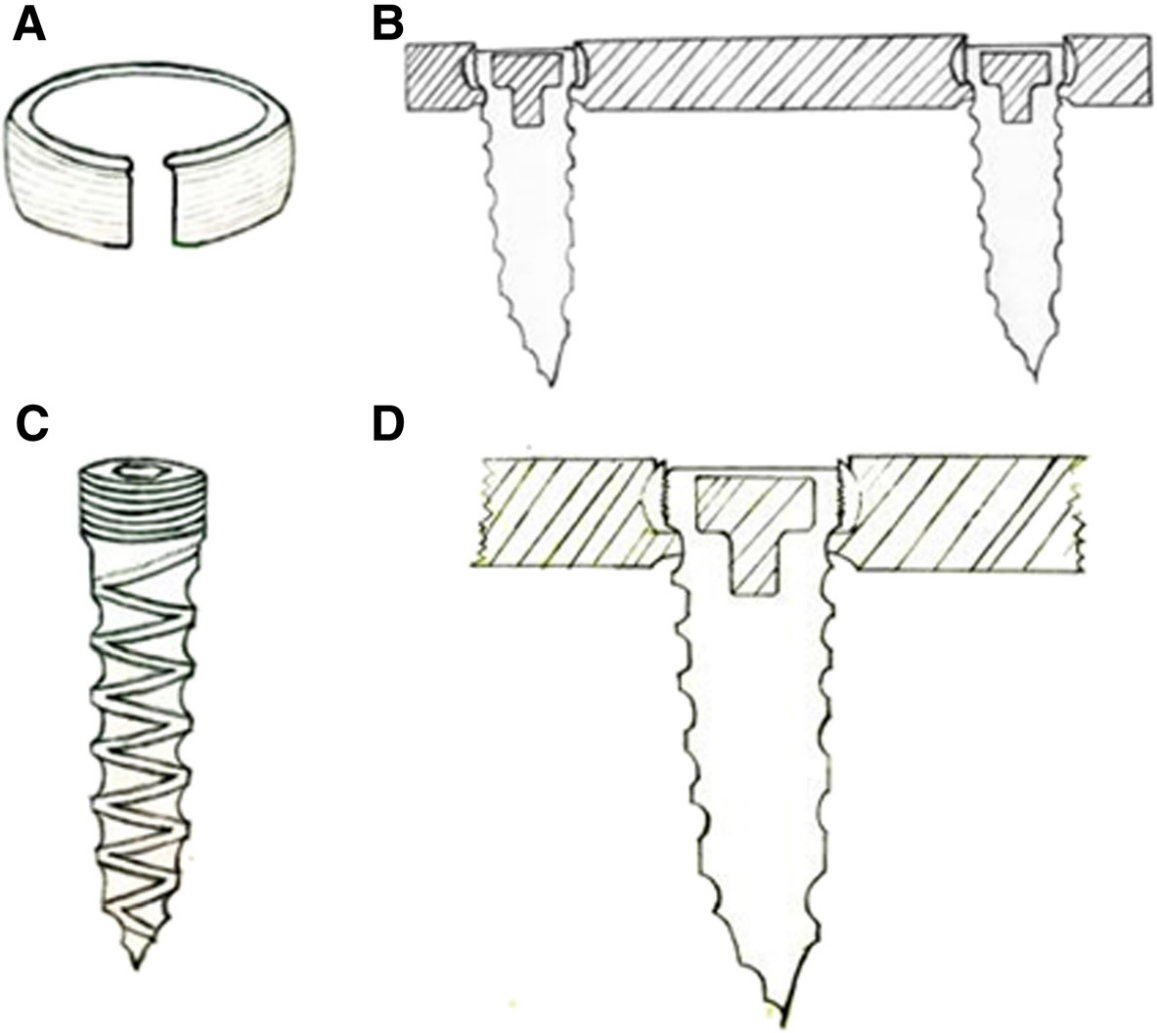
**Screw and ring and the lateral view of the self-locking plate system. (A, C)** The polyethylene ring that is fitted into the screw hole and the screw. **(B, D)** A lateral view of the self-locking plate system, which employs a polyethylene ring to lock the screw into the plate.

A national patent certificate (Patent No.: ZL201220153176.7) was obtained for the novel plate system, including the steel and the screw. The plate was made using a titanium alloy. The key design feature is a polyethylene ring built into the plate’s screw hole. The polyethylene ring has a conical angle of 20° and a range of motion of 15°–30° in the screw hole. The end of the screw has an external thread cycle, and the tail top has an inner six-angle groove, which can be matched with a six-angle screwdriver. The plate and screw length can be modified according to the length requirements of different models (Figure 1). This study was approved by the ethics committee of Ren Ji Hospital, Shanghai Jiao Tong University School of Medicine (approval number: RJ20110034). The fresh adult cadaveric cervical spine specimens were obtained anonymously, and all of them were donated to Anatomy Laboratory, Shanghai Jiao Tong University School of Medicine. So, our ethics committee waived the need for consent from the donor for use of this sample in research.

**Figure 2:**
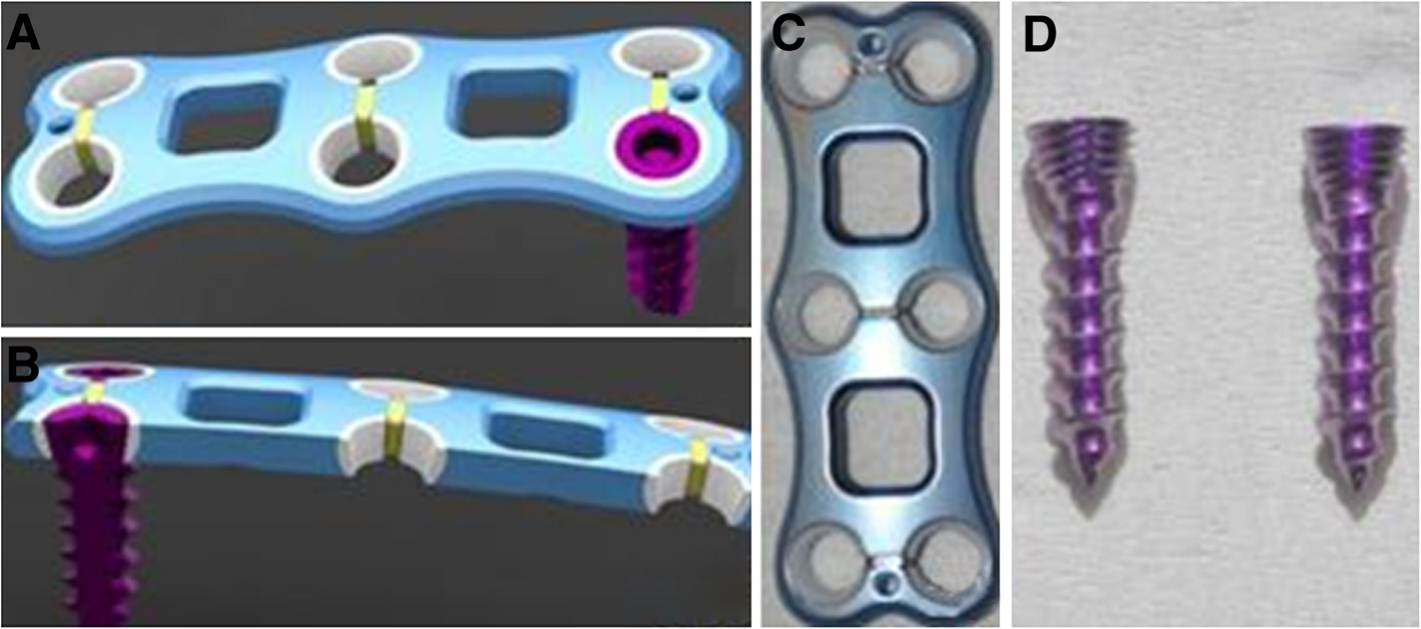
**The self-locking plate system. (A, B)** A schematic diagram of the self-locking plate system. The appearance of the novel self-locking plate **(C)** and screw **(D)**.

In practice, the polyethylene ring was placed into the screw hole, giving the polyethylene ring a conical angle of 20° and a range of motion of 15°–30° in the screw hole. The screws were then screwed into the plate body using a six-angle screwdriver. The external thread cycle of the screw was pressed tightly against with the polyethylene ring in the screw hole, and the corresponding locking thread was cut out. Finally, the screw was locked into the plate and connected into a hole without screw withdrawal or loosening (Figure 2).

### *In vitro* biomechanical testing of the novel self-locking plate system

**Figure 3:**
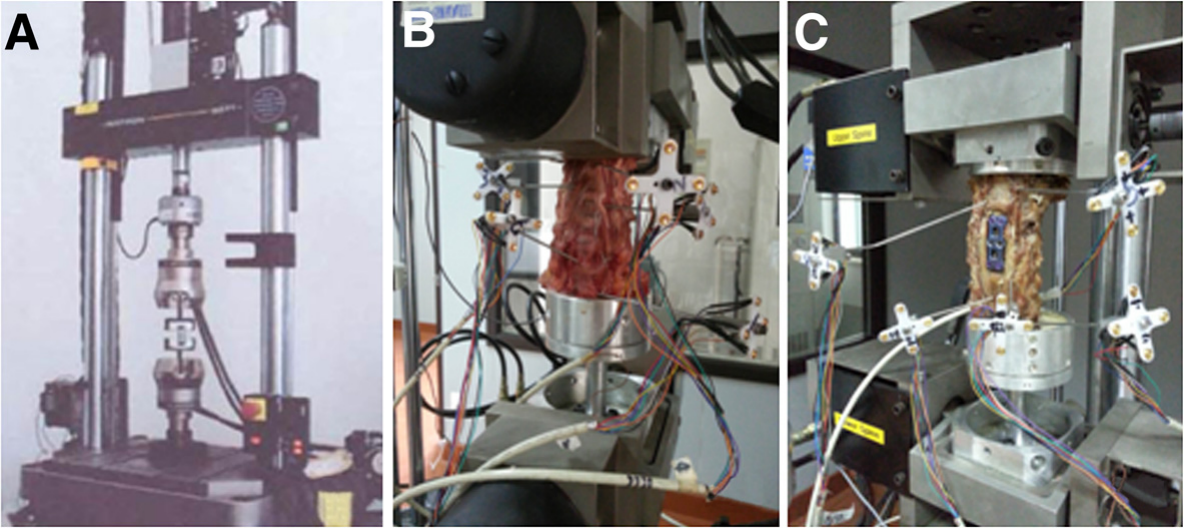
**Experimental setup. (A)** The composite MTS 858 biomechanical testing machine and the test sample. The range of motion, load–displacement, torque, and twisting stiffness tests for the normal specimens **(B)** and the novel self-locking plate fixed specimens **(C)**.

Twelve plates (i.e., six novel plates and six DePuy plates) were subjected to *in vitro* biomechanical testing. The two ends of the plate were fixed to two pieces of a 19-mm module made of ultra-high molecular weight polyethylene, which was designed using the corpectomy model. The test device was connected to the testing machine via a pair of 9.6-mm diameter metal rods that penetrated into the polyethylene module. The test device was subsequently connected to the MTS 858 biomechanical testing machine (Figure 3A). The tensile strength of the polyethylene module used in this experiment was 40 MPa.

The polyethylene module and the plate system were replaced for each pull-out test and fatigue test. The two fixed blocks had an axial rotation around the metal rod during compression and tension, ensuring that the load acted perpendicularly to the fixed block. The experiments were performed at room temperature under dry conditions.

### Pull-out test

The prepared specimens were mounted on the MTS 858 biomechanical testing machine and fixed using a special fixture. The plate and screw system was then subjected to a pull-out test. The maximum pull-out force, the relative displacement required to achieve maximum pull-out force, and the energy absorption value were recorded.

### Fatigue test

**Figure 4:**
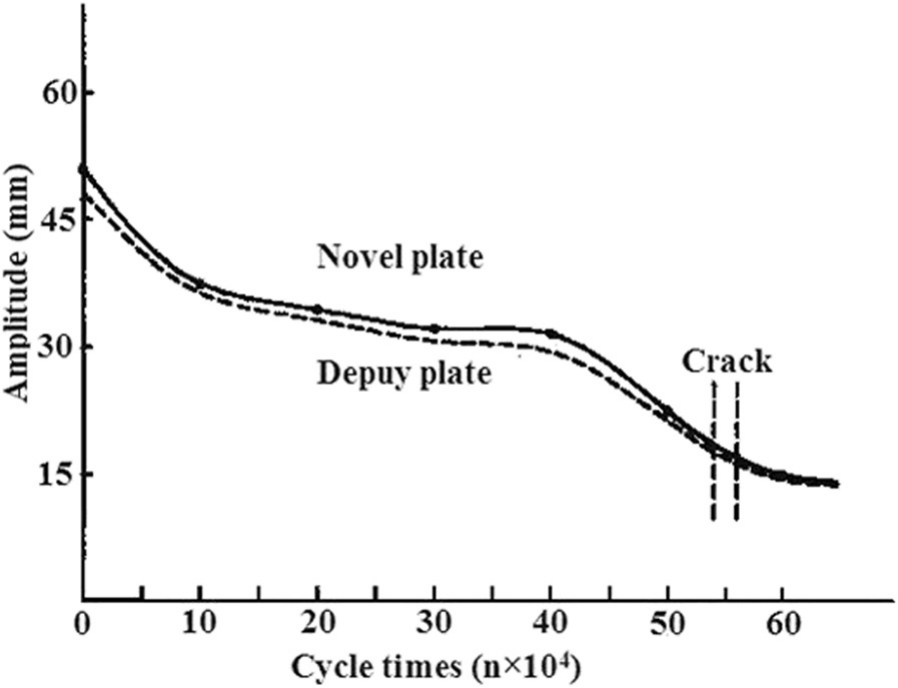
**The fatigue life curves for the novel plate and the DePuy plate.**

A plate that was fixed to the MTS 858 was subjected to a high-frequency fatigue test with sinusoidal loading. The load was 100 N, the loading frequency was 0.5 Hz, the cycle times was set to 106 times, and the load ratio was 10 [6]. All parameters were recorded every 1,000 cycle times, including amplitude and load change. The dynamic stiffness value was obtained. The test was stopped when the plate or the screw exhibited breakage or the cycle times reached 106 times, and the loading frequency or breakage site was recorded. If the fatigue test was completed without breakage, loosening of the plate and the screw was recorded. A fatigue life curve was generated using the experimental data (Figure 4). After the fatigue strength (*σ* − 1) was measured, the maximum stress for the edge of the hole in the titanium plate was calculated using the formula *σ*max = *α* × *σ* − 1, where *α* is the stress concentration factor [7].

### Biomechanical testing of the novel self-locking plate system in cadaveric specimens

Twelve fresh adult cadaveric cervical spine specimens were obtained anonymously. Each specimen included C1-T1, and the average age of the donors was 78 years old, with a range from 71 to 86 years old. Spine specimens with deformities or bone tissue damage were excluded using X-ray radiography. The attached muscle and soft tissue were excised, and the integrity of the ligaments was preserved. To ensure the accuracy of the experiment, the upper and lower ends of the specimen were embedded with polymethyl methacrylate, which formed a loading platform that was within 2° parallel, prior to the experiment. A high-precision grating displacement sensor with clamps was prepared. After measuring the cervical range of motion and the load–displacement, 12 cervical spine specimens (C1-T1) were subjected to anterior cervical corpectomy and C5 fusion (ACCF) using six novel plates and six DePuy plates under randomized double-blind conditions. Titanium mesh with an autogenous bone graft was implanted. The cervical plates in the two groups had the same length. The fixation of all specimens was confirmed using X-ray radiography.

### Pre- and postoperative measurements of cervical range of motion, load–displacement, axial stiffness, torque, and twisting stiffness

The specimen was mounted on an MTS 858 biomechanical testing machine, the sensor was connected (Figure 3B, C), and the center of gravity and loading point were confirmed. An average weight of 150 N for the general head load was set as the experimental load, and the torque was 2 Nm [6]. Vertebral creep and relaxation time effect were eliminated by preloading before the experiment. The cervical range of motion, axial compression, flexion, extension, left buckling, right buckling, relative displacement, relative torsional angle, and torsional torques were measured. Axial stiffness and torsional stiffness were calculated using the previously described formula [7]. Axial stiffness (EF) refers to the axial deformation resistance ability of the cervical spine under axial load. The formula used to calculate axial stiffness is EF = *PL*/*△L*, where *P* is the load, *L* is the cervical vertebral height, and *△L* is the relative displacement of the cervical spine. The mechanical definition of torsional stiffness refers to the capacity to resist torsional deformation. The formula used to calculate torsional stiffness is *GJp* = Mn/*θ*, where Mn is the torque and *θ* is the torsion angle.

### Statistical analysis

The statistical significance was calculated using the paired *t*-test. The data were analyzed using the SPSS for Windows software program, version 19.0. All significance levels were set at *P* <0.05.

## Results

### *In vitro* biomechanical testing of the novel self-locking plate system

No significant differences in maximum pull-out force, relative displacement required to achieve maximum pull-out force, or energy absorption value were observed between the novel plate and the DePuy plate (*P* >0.05), whether the complete screw-plate system or only the screw was tested (Table 1). This finding suggests that the pull-out strength was equivalent for the two types of plates. In the fatigue test, screw loosening was observed first, followed by plate cracking, which increased until breakage occurred. The fatigue life of the two types of plates was similar. The cycle times for the novel plate and the DePuy plate were 5.6 × 10^5^ and 5.4 × 10^5^, respectively. The fatigue strength of the two types of plates was also similar. The strength of the novel plate was 490.75 MPa, and the strength of the DePuy plate was 485.86 MPa (Table 2). The fatigue curve is presented in Figure 4. As the load increased, stress increased and fatigue life was shortened. Under high stress, the plate broke at both ends of the screw hole. The break was characterized as a critical brittle fracture that developed from fatigue crack extension. The fracture cracks were derived from minus cracks at the hole edge, which gradually extended and expanded until breakage occurred. In addition, the fatigue strength (*σ* − 1) of the novel plate was 490.75 MPa. According to the formula *σ*max = *α* × *σ* − 1, the maximum stress of the novel plate at the edge of the hole was *σ*max =628.16 MPa, while the maximum stress of the DePuy plate at the edge of the hole was 621.90 MPa.

**Table 1:** **Comparison of pull-out test between novel self-locking plate and DePuy plate (** ***P*** **>0.05)**

**Parameters**	**Screw-plate whole system**		**Screw only system**	
	**Novel plate**	**DePuy plate**	**Novel plate**	**DePuy plate**
Maximum pullout force (N)	394.55	389.76	238.65	230.76
Relative displacement (mm)	12.34 ± 1.05	12.12 ± 1.26	11.88 ± 1.12	11.25 ± 0.96
Energy absorption value (J)	6.25 ± 1.22	5.97 ± 1.56	2.75 ± 1.35	2.52 ± 1.26

**Table 2:** **Comparison of fatigue test between novel self-locking plate and DePuy plate**

	**Load range (N)**	**Frequency (Hz)**	**Cycle times (** ***n*** **× 10** ^**6**^ **)**	**Fatigue strength (MPa)**	**Damage**
Novel plate	100 ~ 150	0.5	0.56	490.75	Screw loose, crack at outer edge of right upper hole
DePuy plate	100 ~ 150	0.5	0.54	485.86	Screw loose, crack at outer edge of right upper hole

### Biomechanical testing of the novel self-locking plate system in cadaveric specimens

**Figure 5:**
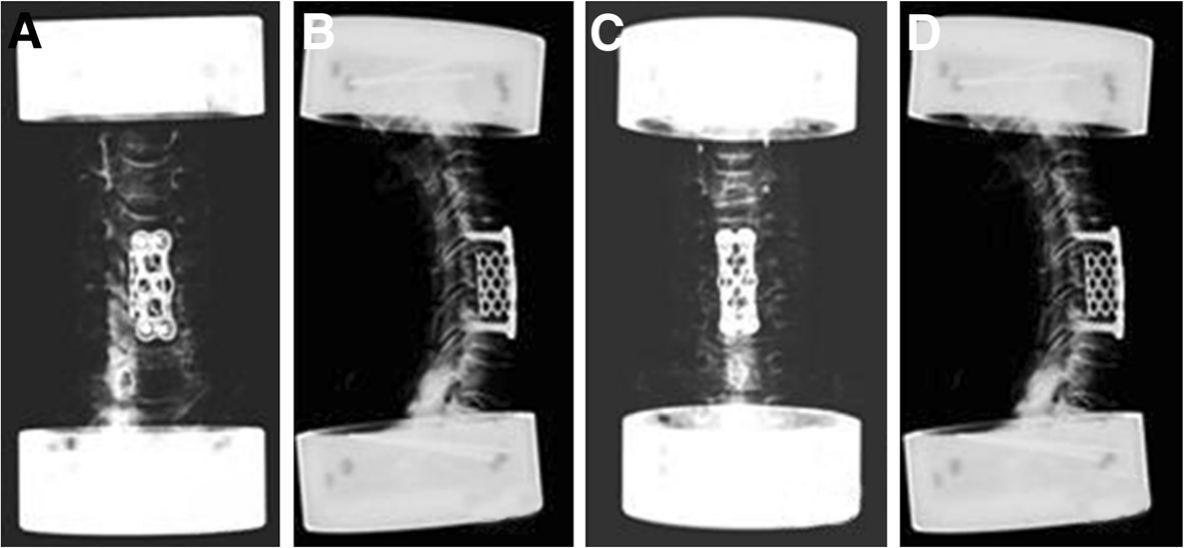
**Postoperative anteroposterior and lateral radiographs of the anterior cervical corpectomy and the fusion of C5. (A, B)** The novel self-locking plate system and **(C, D)** the DePuy plate system.

Comparison of the cervical range of motion of the two fixations (Figure 5). No significant difference in cervical range of motion was observed between the two anterior cervical plates. The novel plate did not restrict cervical mobility more than the DePuy plate (Table 3).

**Table 3:** **Comparison of cervical range of motion between the two fixations (mean ± SD) (degrees) (** ***P*** **>0.05)**

**Position**	**Level**	**Novel plate**	**DePuy plate**
Flexion	Total motion	26.45 ± 1.25	24.43 ± 1.77
	C3-C4	4.35 ± 0.78	4.13 ± 0.55
	C6-C7	4.76 ± 0.68	4.69 ± 0.65
Extension	Total motion	20.85 ± 1.12	21.88 ± 1.35
	C3-C4	4.49 ± 1.98	4.23 ± 0.71
	C6-C7	4.53 ± 1.28	4.62 ± 1.84
Left buckling	Total motion	33.07 ± 1.22	32.95 ± 1.58
	C3-C4	5.49 ± 1.18	5.37 ± 0.95
	C6-C7	5.68 ± 1.58	5.58 ± 1.15
Right buckling	Total motion	35.52 ± 1.56	37.55 ± 0.97
	C3-C4	5.62 ± 0.79	5.54 ± 0.67
	C6-C7	5.75 ± 0.85	5.68 ± 1.03
Left rotation	Total motion	18.67 ± 0.83	18.48 ± 0.58
	C3-C4	3.12 ± 0.54	3.02 ± 0.71
	C6-C7	3.32 ± 0.84	3.26 ± 0.46
Right rotation	Total motion	18.82 ± 0.93	18.51 ± 0.89
	C3-C4	3.42 ± 0.45	3.38 ± 0.57
	C6-C7	3.52 ± 0.83	3.60 ± 0.55

**Figure 6:**
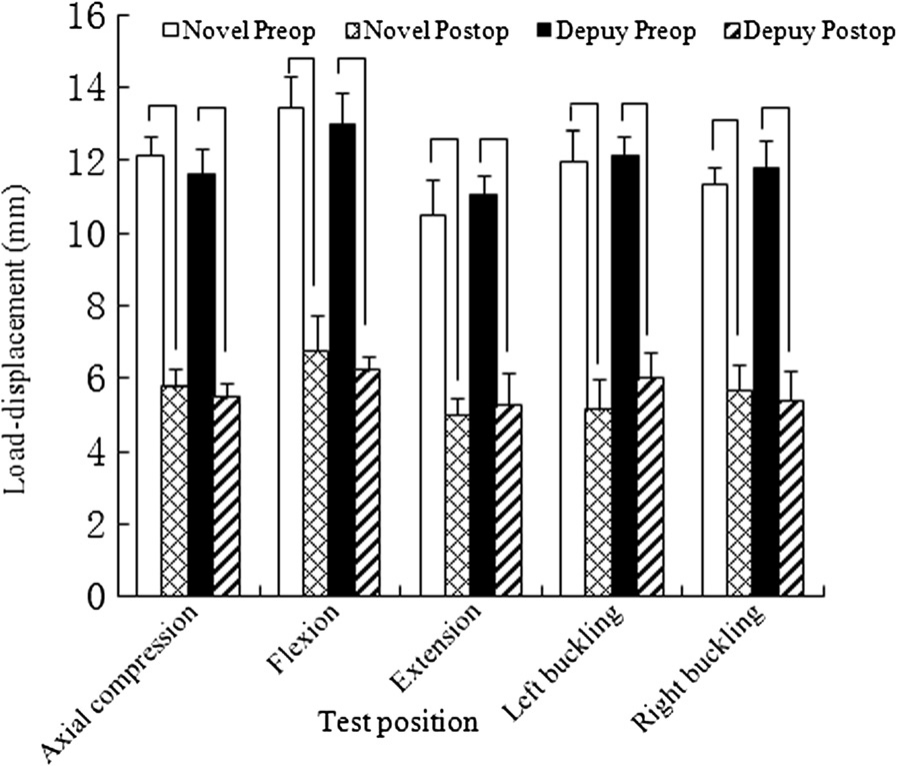
**A comparison of cervical load–displacement between the two plate groups.** No significant difference in normal cervical longitudinal displacement was observed between the two plate groups (*P* >0.05). Significant differences in longitudinal displacement for five types of motion were observed between preoperative spines and both groups of postoperative internally fixed spines (*P* <0.05).

Comparison of the cervical load–displacement of the two fixations. No significant difference in normal cervical longitudinal displacement was observed between the two plate groups (*P* >0.05). This result indicated that the variation between the two groups was acceptable and that the longitudinal displacement of a normal cervical spine could be used as a control parameter for the two plate groups (Table 4). Significant differences in longitudinal displacement for five types of motion were observed between normal cervical spines and cervical spines that were internally fixed using both types of plates (*P* <0.05) (Figure 6). No significant differences in displacement decrease or longitudinal displacement after internal fixation were observed between the two plate groups. Thus, both types of internal fixation significantly reduced the longitudinal displacement of the cervical spine and increased stability.

**Table 4:** **Comparison of cervical load–displacement between the two fixations (mean ± SD) (mm) (** ***P*** **>0.05)**

**Position**	**Novel plate (** ***n*** **=6)**		**DePuy plate (** ***n*** **=6)**	
	**Preoperation**	**Postoperation**	**Preoperation**	**Postoperation**
Axial compression	12.12 ± 0.55	5.75 ± 0.48	11.65 ± 0.65	5.45 ± 0.42
Flexion	13.45 ± 0.87	6.73 ± 0.98	12.97 ± 0.85	6.21 ± 0.35
Extension	10.52 ± 0.92	4.98 ± 0.44	11.04 ± 0.53	5.25 ± 0.88
Left buckling	11.96 ± 0.85	5.12 ± 0.82	12.15 ± 0.49	5.96 ± 0.74
Right buckling	11.32 ± 0.48	5.65 ± 0.73	11.78 ± 0.75	5.38 ± 0.83

Comparison of the torque and twisting stiffness of the two fixations. When the load was 150 N, the cervical axial stiffness of the cervical spine with subtotal corpectomy was 32 N/mm, the cervical axial stiffness of the cervical spine with titanium mesh and self-locking plate fixation increased to 45 N/mm, and the cervical axial stiffness of the cervical spine with titanium mesh and DePuy plate fixation increased to 43 N/mm. No significant difference was observed between the two plates (*P* >0.05). Thus, both kinds of anterior cervical locking plate fixation significantly enhanced the ability of the cervical spine to resist deformation, mediating similar increases in cervical stability.

No significant differences in torque or torsional stiffness were observed between the two plate groups when using the same loads and relative torsional angles (*P* >0.05) (Table 5). Thus, cervical resistance to torsional deformation after both types of internal fixation was similar, indicating that both types of cervical plates provide equivalent torsional stability after fixation.

**Table 5:** **Comparison of torque and twisting stiffness between the two fixations (mean ± SD) (** ***P*** **>0.05)**

	**Torque (Nm)**	**Twisting stiffness (Nm × cm/D)**
Novel plate	2.84 ± 0.12	21.46 ± 1.24
DePuy plate	2.78 ± 0.08	21.15 ± 1.43

## Discussion

The development of the economy and modern medical advances has prolonged the average life span, and the incidence of senile degenerative cervical spondylosis, cervical injury, and cervical vertebral metastatic tumors increases each year. In addition to providing pain relief via decompression of the spinal cord or nerve root, it is important to rebuild the stability of the spine [8]. Since Bohler and Ganderhak [9] first used AO plates and screws for anterior cervical fixation, the anterior cervical plate system has experienced nearly half a century of development and improvement and is widely used in patients with cervical spine trauma, tumors, and multi-segment degeneration. The current most frequently used locking plate requires tightening of the locking nut after the screw is tightened. One disadvantage of this design is increased operative time. The locking device also occupies a portion of the plate and bone viewing window. Bone graft healing may also be affected. We analyzed international development trends for cervical plates and developed an anterior cervical self-locking plate system.

Osteopenic samples were used in this study because the samples were harvested from elderly and systemically ill donors (e.g., rheumatoid arthritis) [10,11]. Using these samples, we were able to assess the stability and tightening of the implanted plate system in the elderly. This study model has been described in previous studies [12-16].

This plate system was constructed using a government-approved medical titanium alloy that is lightweight with good biocompatibility, high corrosion resistance, low stress shielding, and 80%–90% of the strength of steel. The length of the plate can be modified to meet different clinical needs. The plate was prebent to match the physiological curvature of the cervical spine, and the curvature could also be adjusted intraoperatively. The polyethylene ring had a conical angle of 20° and a range of motion of 15°–30° in the screw hole. When the external thread cycle of the screw was pressed tightly against the polyethylene ring in the screw hole, the corresponding locking thread was cut out. The screw was then locked into the plate and connected into a hole without screw withdrawal or loosening. The advantages of the new plate system include simple structure, convenient operation, a good operative view, and multiple flexible screwing directions. The low-notch plate hole design reduces postoperative adjacent intervertebral ossification. This anterior cervical self-locking system is stable, allowing clinicians to reduce operative time, improve the quality of the operation, and reduce the pain of the patients.

The currently used method of biomechanical evaluation was introduced by Panjabi [17]. The biomechanical properties of a spinal internal fixation instrument include two main factors. First, the mechanical properties of the instruments, such as the equipment fatigue strength, must be determined in the physiological environment by assessing the equipment using the strength test and the fatigue test under a variety of physiological loads. Second, the fixing effect of the devices on an unstable spine must be assessed using the stability test [7]. Real physiological loading of the cervical spine was not accurately represented because preloading did not occur and muscle forces were neglected. However, because the physiological load is unknown, the loading conditions used in this test are widely accepted and facilitate standardized testing [18-21].

No significant differences in the pull-out test were observed between the novel plate and the DePuy plate, whether the complete screw-plate or only the screw was used. The novel plate performed well with respect to fastening, screw fixation, and integral locking. The fatigue test indicated that the hole edge of the novel plate would rupture when the fatigue endurance reached 628.16 MPa. The outside of the hole edge cracked and gradually extended, and rupture occurred with a load of 10^6^ cycle times. Therefore, the area surrounding the screw hole, particularly the outer edge, experienced the most concentrated stress. This phenomenon suggested potential improvements for the novel plate. Any anterior cervical plate system is required to open the hole for fixation of the vertebral body and the bone graft. This process leads to stress concentration around the hole and results in fatigue fracture. It is crucial for the plate system to have sufficient strength to withstand frequent loading over a long period of time. Because the novel plate system can withstand an average of 5.5 × 10^5^ or more times the load before cracking, the fatigue strength is more than 490 MPa. This fatigue strength is sufficient to improve postoperative cervical stability after bone graft fusion, and the plate system would be safe and effective for a long period of time *in vivo*.

This study aimed to compare the effects two types of anterior cervical locking plates on the stability of the cervical spine. Biomechanical testing confirmed that the novel anterior cervical self-locking system had good strength and fastening ability and could be used for more convenient reconstruction of cervical stability. The limitation of this study was the sample size, which was limited to 12 plates and 12 specimens. Another common limitation of anterior cervical plate is the high rate of pseudarthrosis and hardware breakage. Therefore, this novel plate system requires further clinical tests in human subjects. The preliminary experimental results of this study demonstrated that this novel design is beneficial for both surgeons and patients.

## Conclusions

Compared to the DePuy plate, the novel anterior cervical self-locking plate system described here has good strength and fastening ability, allowing it to provide sufficient biomechanical stability. Further clinical assessment of this system is needed.
